# Straight Longitudinal Aortotomy for Aortic Valve Replacement Through Right Anterior Minithoracotomy

**DOI:** 10.1016/j.atssr.2025.05.011

**Published:** 2025-06-06

**Authors:** Masaya Nakamizo, Naonori Kawamoto, Satoshi Kainuma, Kota Suzuki, Takashi Kakuta, Masaya Hirayama, Kohei Tonai, Satsuki Fukushima

**Affiliations:** 1 Department of Cardiac Surgery, National Cerebral and Cardiovascular Center, Suita, Japan

## Abstract

Conventional aortotomy in aortic valve replacement (AVR) is transverse or oblique. However, traditional aortotomy is difficult in patients with a high takeoff right coronary artery. Studies have reported aortotomy techniques for AVR through sternotomy, but not through right anterior minithoracotomy. We report 6 patients who underwent AVR by means of right anterior minithoracotomy through a straight longitudinal aortotomy, which provided good aortic valve exposure and facilitated hemostasis. The procedure was successfully performed without major complications, with favorable postoperative outcomes. This technique may be a viable alternative to traditional aortotomy, thus offering improved surgical field visibility and ease of closure.

Minimally invasive cardiac surgery has been steadily developing and is increasingly performed. Some studies have demonstrated that using the right anterior minithoracotomy (RAT) approach for aortic valve replacement (AVR) yields superior postoperative results compared with conventional sternotomy.^1^^,^^2^ In general, a transverse or oblique incision is preferred for both RAT AVR and sternotomy AVR. However, it could be challenging to perform RAT AVR using a transverse or oblique aortotomy in patients with a high takeoff of the right coronary artery or distribution of calcification in the ascending aorta and sinotubular junction (STJ). Some studies have reported the use of aortotomy techniques for sternotomy AVR.^3^, ^4^, ^5^ Here we present 6 cases in which a straight longitudinal aortotomy was used for RAT AVR.

Patient 1 was a 69-year-old man with severe aortic regurgitation (AR) who had a high takeoff of the right coronary artery, which branched from the proximal ascending aorta 2 cm above the STJ (Figure 1A). Patient 2 was an 80-year-old woman with tricuspid aortic valve stenosis (AS). Patient 3 was an 83-year-old man with severe AR. Patient 4 was a 75-year-old woman with tricuspid AS. Patient 5 was a 76-year-old woman with bicuspid AS who had a mildly dilated ascending aorta of 41 mm. Patient 6 was a 77-year-old woman with tricuspid AS who had patchy calcification in the ascending aorta and STJ (Figure 1B). All patients underwent RAT AVR with a straight longitudinal aortotomy, as described later. The mean operation time, cardiopulmonary bypass time, and cardiac arrest time were 197, 120, and 76 minutes, respectively. No patients had major complications. Postoperative echocardiography showed good cardiac function without paravalvular leakage greater than mild in all patients. All patients were discharged to their homes within 3 weeks postoperatively.

**Figure 1:**
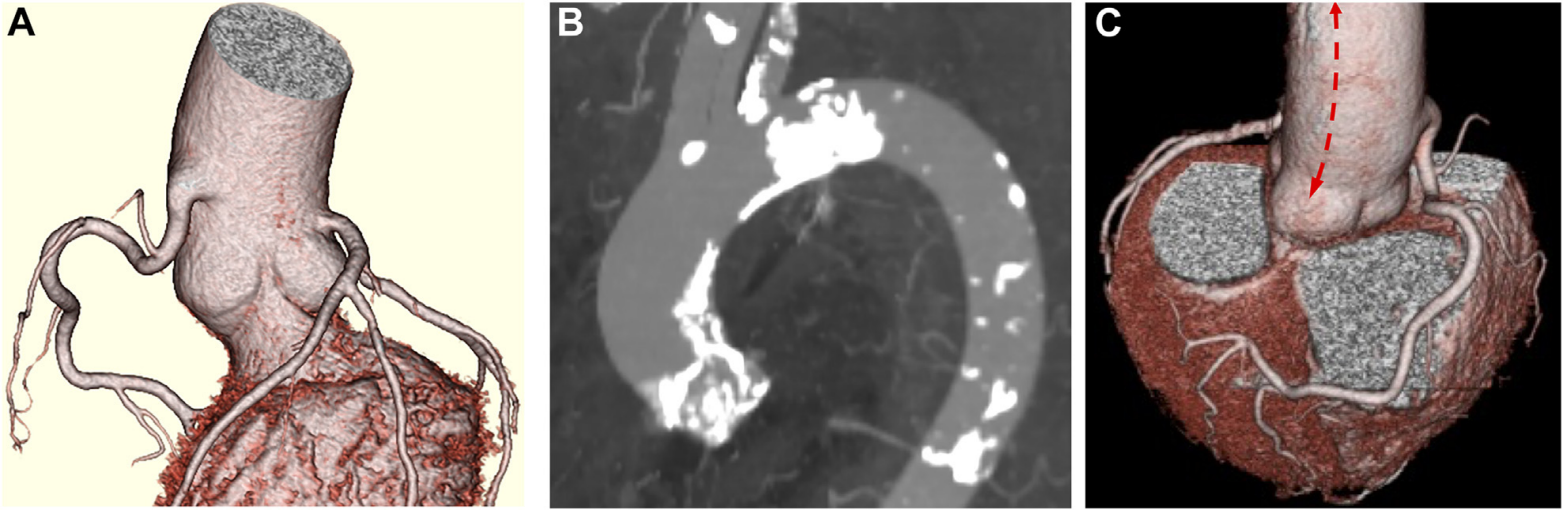
(A) Preoperative 3-dimensional reconstructed contrast-enhanced computed tomographic image showing the high takeoff of the right coronary artery in patient 1. (B) Distribution of calcification in the ascending aorta and sinutubular junction of patient 6. (C) The straight longitudinal aortotomy line (dashed red line with arrows) on a preoperative 3-dimensional reconstructed contrast-enhanced computed tomographic image of patient 1.

In our procedure, the patient was placed in the partial left lateral position with approximately 30° rotation from the spinal position. RAT AVR was performed by means of a 5- to 7-cm incision through the right third intercostal space (Figure 2). A camera port with a carbon dioxide infuser was inserted into the second intercostal space. Cardiopulmonary bypass was initiated through the right femoral artery, right femoral vein, and right internal jugular vein. A root cannula was inserted into the ascending aorta for antegrade cardioplegia, venting, and pressure monitoring. A vent tube was inserted through the right superior pulmonary vein. The distal ascending aorta was cross-clamped, and cardioplegia was infused through the root cannula. After cardiac arrest, a 6- to 8-cm straight longitudinal incision was made from the lateral aspect of the ascending aorta 3 to 5 cm below the origin of the brachiocephalic artery to just above the nadir of the noncoronary cusp, as guided by preoperative computed tomographic imaging (Figure 1C). Traction sutures were then placed on the anterior and posterior aspects of the aortotomy to allow retraction of the aorta and better exposure of the aortic valve (Figure 3A). After removing the aortic valve leaflet, AVR was performed using a tissue aortic valve in the supraannular position with noneverting mattress sutures (Figure 3B). The aortotomy was closed in 2 layers (Figure 3C, Video). After the aortic cross-clamp was removed, weaning from cardiopulmonary bypass was uneventful in all cases.

**Figure 2:**
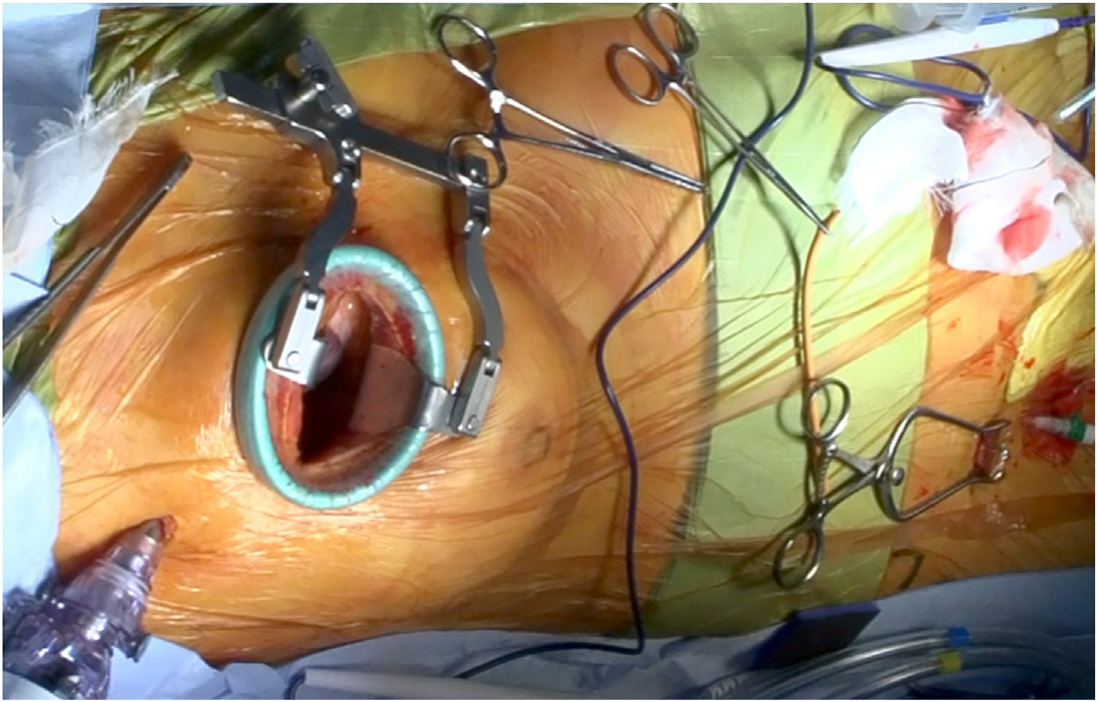
Surgical field setting in patient 2.

**Figure 3:**
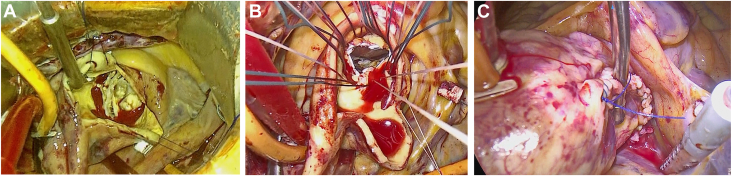
(A) Surgical vision of the aortic valve from the incision site in patient 5. (B) Noneverting mattress suture on the aortic annulus of patient 3. (C) Closed incision line seen from the camera port in patient 3.

## Comment

We consider that this straight longitudinal incision for RAT AVR has some advantages over the traditional transverse or oblique incision regarding visualization of the coronary artery, aortic valve exposure, and hemostasis of the closure line.

In patients with a high takeoff right coronary artery such as patient 1, previous studies have reported the use of a modified transverse or oblique aortotomy for sternotomy AVR.^3^, ^4^, ^5^ However, there are no reports of the aortotomy technique used in RAT AVR. It may be technically challenging to perform RAT AVR with a traditional transverse or oblique aortotomy in patients with a high takeoff right coronary artery because of coronary artery interference and the limited surgical field. This straight longitudinal incision starts from the lateral side of the ascending aorta and extends straight down to the nadir of the noncoronary cusp, which means that this incision passes far from both coronary ostia. Therefore, there is no need to pay special attention to prevent interference with the coronary artery, even though the right coronary artery arises from the anterior ascending aortic wall.

In patients with a calcified ascending aorta and STJ, some surgeons may hesitate to plan RAT AVR with a traditional transverse or oblique aortotomy. However, if the lateral wall of the ascending aorta and STJ remain uncalcified, RAT AVR with a straight longitudinal aortotomy is feasible, as seen in patient 6.

In all 6 cases, the aortic annulus was exposed with good visibility through a straight longitudinal aortotomy. By placing the traction sutures on the anterior and posterior aspects of the longitudinal aortotomy, the STJ and Valsalva sinus could be widely opened, thereby enabling good visualization of the whole leaflet and aortic annulus (Figures 3A, 3B). It can be demanding to resect and decalcify the aortic valve when access is limited and the root anatomy is small. In this situation, an extension of the aortotomy and placement of additional aortic wall or commissural stay sutures may improve visualization of the entire annulus.

Closing the straight longitudinal aortotomy was easy and safe compared with a transverse or oblique aortotomy because the whole incision line was close to the skin incision (Figure 3C). Consequently, hemostasis of this aortotomy may be easier to complete. The mean cardiac arrest time, cardiopulmonary bypass time, and operation time in our report were relatively short compared with the values for isolated RAT AVR reported by the Japanese registry,^6^ and our findings may support our perspective that the straight longitudinal aortotomy provides better valve exposure and easy hemostasis.

In conclusion, we successfully performed RAT AVR using a straight longitudinal aortotomy, which provides good exposure and easy hemostasis of the closure line. More cases need to be accumulated to verify the utility of this aortotomy technique.

## Supplementary Data

Video Surgical video from aortotomy to closure in patient 5.
